# Infiltration Route Analysis Using Thermal Observation Devices (TOD) and Optimization Techniques in a GIS Environment

**DOI:** 10.3390/s100100342

**Published:** 2010-01-04

**Authors:** Soonam Bang, Joon Heo, Soohee Han, Hong-Gyoo Sohn

**Affiliations:** 1 Agency for Defense Development, P.O. Box 35 Yuseong, Daejeon, Korea; E-Mail: snbang@hanafos.com; 2 School of Civil and Environmental Engineering, Yonsei University, 262 Seongsanno, Seodaemun-gu, Seoul 120-749, Korea; E-Mails: jheo@yonsei.ac.kr (J.H.); scivile@yonsei.ac.kr (S.H.)

**Keywords:** thermal observation device, infiltration-route analysis, dynamic simulation, GIS, A* algorithm

## Abstract

Infiltration-route analysis is a military application of geospatial information system (GIS) technology. In order to find susceptible routes, optimal-path-searching algorithms are applied to minimize the cost function, which is the summed result of detection probability. The cost function was determined according to the thermal observation device (TOD) detection probability, the viewshed analysis results, and two feature layers extracted from the vector product interim terrain data. The detection probability is computed and recorded for an individual cell (50 m × 50 m), and the optimal infiltration routes are determined with A* algorithm by minimizing the summed costs on the routes from a start point to an end point. In the present study, in order to simulate the dynamic nature of a real-world problem, one thousand cost surfaces in the GIS environment were generated with randomly located TODs and randomly selected infiltration start points. Accordingly, one thousand sets of vulnerable routes for infiltration purposes could be found, which could be accumulated and presented as an infiltration vulnerability map. This application can be further utilized for both optimal infiltration routing and surveillance network design. Indeed, dynamic simulation in the GIS environment is considered to be a powerful and practical solution for optimization problems. A similar approach can be applied to the dynamic optimal routing for civil infrastructure, which requires consideration of terrain-related constraints and cost functions.

## 1. Introduction

Routing analysis has been one of the most popular research topics in geospatial-related fields. A variety of computing techniques have been developed using different datasets, and the performances of routing analysis techniques for determining optimized routes for infrastructure facilities (e.g., gas pipelines) [1,2] and transportation-related projects [3–5], based on terrains and other terrain-related constraints, have been tested and improved. A routing algorithm based on neural network techniques was even applied to real-time forecasting of stream flows during storms for effective operational flood management [6]. Geospatial information has been widely used for military applications as well, and its importance in modern warfare is considered to be very valuable. It has become possible to analyze the terrain conditions of inaccessible areas with various sources of data collection sensors. Moreover, the increased spatial and spectral resolutions of sensors support more detailed analyses of terrain conditions. Therefore, geospatial information has been a primary resource for various military tactical applications, such as mission planning, terrain-contour matching for cruise-missiles and aircraft, viewshed analyses, infiltration-route analysis, impact – point analysis, hidden-point analysis, and urban–battle analysis [7–11].

Path planning is one of the primary research topics in many application fields, and a variety of methods have been used to find optimal routes [12,13]. Depth-first search and breadth-first search methods often require a huge amount of processing time. The greedy methods sometimes can be impractical for obtaining optimal solutions. Dijskstra’s algorithm [14,15] is a widely used method to find optimal solutions in path-searching analysis, and A* algorithm [16] can provide approximated optimal solutions with even less computing time.

Meng [9] used both A* and Dijkstra’s algorithms to determine optimal paths, and found that a raster-based route analysis method required processing of an increased amount of data and a huge amount of computing time. Marti *et al.* [17] employed commercial ESRI software for raster-based path planningusing high-resolution terrain data and other information. Dynamic programming approach is applied to raster-based probability maps which illustrate movable and non-movable regions to determine the optimal paths of mountainous areas [18]. Helgason *et al.* [11] analyzed optimal routes in a static environment using the Voronoi diagram. John *et al*. [10] applied integer programming and a genetic algorithm to path-searching on a rectangular grid to optimize the local surveillance planning. Howard *et al.* [19] computed the optimal paths for planetary rovers by applying the A* algorithm to raster-based fuzzy maps containing five terrain categories. Niederberger [20] proposed a new algorithm, a revised A* algorithm, that could find routes instinctively in a fast and stable manner. Saha *et al.* [21] produced a thematic cost map in raster form, and applied Dijkstra’s algorithm for optimal routing between fixed origin and destination in landslide-prone area. Smith [22] used horizontal and vertical curvature constraints to determine the shortest gradient-constrained paths across terrain surfaces. These studies proved that raster-based probability maps could present an appropriate environment for static-environment infiltration-route analyses with surveillance equipment. Caccetta *et al.* [23] dealt with determining an optimal transit path for a submarine moving by minimizing the overall probability of detection through a field of sonar sensors.

Most current studies for optimal path determination are in the civilian use and not many military applications can be found. For military application thermal observation devices (TODs) combined with a heuristic approach based on Dijkstra’s algorithm are utilized to solve a constrained path problem in this study. TOD is a night vision system which can detect and quantize the thermal energy radiated from moving objects. The objective of this study is to present a solution for optimal infiltration-route analysis with TODs in a geospatial information system (GIS) environment and then to confirm its validity for computationally intensive simulation environment. To those ends, this paper presents a computational environment for infiltration-route analysis that can discover infiltration vulnerability. Among the possible shortest-path-searching algorithms, the A* algorithm was implemented to determine susceptible routes based on terrain features and surveillance networks. In the simulations, the surveillance networks employed TODs. An imaginary military demarcation line (MDL) was established, and TODs were virtually positioned inside the infiltration field. Several raster-based detection probability maps were created using various terrain information pertaining to concealment and detection. The intermediate results were then merged together to create a final detection probability map. The objective function minimized the summed detection probability, or in other words, the cumulative detection probability on the routes. Then, a shortest-path-searching algorithm was applied to the final detection probability map, and the optimal infiltration routes from start point were identified. In order to reflect a dynamic real-world problem, the same approach was repeatedly applied one thousand times while the location of the TODs and start points were randomly changed under certain constraints. The accumulation of optimal infiltration routes can achieve an infiltration vulnerability map. This map can be used as an input layer in designing the optimal TOD locations from a surveillance perspective, which also requires simulation to maximize detection probability. Those results would indicate the value of dynamic simulation to the optimization problem as well as the value of the GIS framework to the solution of such complicated routing problems.

## 2. Overview of Data and Methodology

### 2.1. Description of Input Data

**Figure 1.**
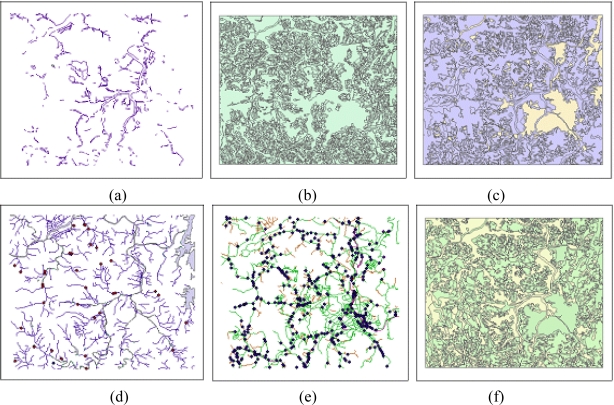
VITD’s Six Layers. (a) Obstacle (OBS). (b) Topography (SLP). (c) Soil (SMC). (d) Irrigation (SDR). (e) Transportation (TRN). (f) Vegetation (VEG).

VITD, DEM, and TOD detection probabilities were used to create detection probability maps in this study. Defined by the National Geospatial-Intelligence Agency (NGA) military standard [24], VITD is a geospatial information standard containing exhaustive terrain information for military operations. Figure 1 shows six different layersof obstacles (OBS), slope/surface configurations (SLP), soil/surface materials (SMC), surface drainage (SDR), transportation (TRN), and vegetation (VEG) included in VITD data. The OBS coverage includes natural obstacles and man-made obstacles. The SLP coverage illustrates terrain objects of similar slope ranges. The unit of slope is the percent slope, and the minimum area is 5,000 m^2^ (50 m × 100 m). The SMC coverage characterizes a soil classification system showing soil groups based on rock outcrops, permanent snowfields, evaporation, and other factors. The SDR coverage contains information on rivers, canals, hydrology, dams, lakes and various other bodies of water. The TRN coverage represents terrain attributes associated with different forms of transportation including roadways, railways, bridges, tunnels, and airfields. The VEG coverage illustrates military-application-related landuse and various types of vegetation. The TRN, SLP and SMC layers can be used to evaluate the movement of vehicles, whereas the SDR coverage can be used to select hiding places [8]. VEG layer was used to compute concealment probability related to tree occlusions in this study.

A high-resolution DEM is preferable to precisely depict real terrain. Therefore, a DEM of 5 m × 5 m cell size was created from the contour lines on a 1:5,000 scale Korean digital topographic map. The TOD surveillance locations are normally situated at positions with fully-open views to the front. Twelve TODs were assumed in this study, and their locations were determined according to terrain conditions, which will be discussed in detail in Section 3. The TOD detection probability was computed using the ACQUIRE model, as mentioned previously. The ACQUIRE model presents the detection probability for a given TOD with respect to distance, which is estimated through repetitive iterations.

A large number of detection probability maps were created using concealment probability, viewshed analysis, and TOD detection probability. Concealment probability maps represent the probability of infiltration exposure based on terrain occlusions; viewshed analyses compute the visible area from TOD locations according to terrain occlusions and line of sight; and TOD detection probabilities imply computed viewing probability of all regions from TOD locations.

Raster-based 50 m × 50 m probability maps were created using ArcGIS 9.0. Each cell has a detection probability value sensed by TOD, with regard to all of the associated features. The area of experimentation was 20 km by 13 km, and the total number of nodes represented in the form of graph data structure was 104,000 (400 × 260). Map projection for all of the spatial data was UTM Zone 52 on the datum of WGS84.

### 2.2. Thermal Observation Device (TOD)

TOD is a thermal imaging system which measures infrared energy emitted from a body with temperature. It extends our vision beyond the short-wavelength red into the far infrared by making the light, emitted by warm object, naturally visible [25]. It is also widely used in such areas as detection of energy loss from a building, measurement of storage quantity inside of a store tank, confirmation on abnormality of electrical transmission line, and lookout for trespassers. Recently, it finds its applications in the analysis and inspection of a printed circuit board, atmospheric survey by satellites, medical appliances, and so on.

In military applications, the thermal imaging system is mainly used as an observation device for the precise identification of targets during the night. The true value of thermal imaging was well demonstrated during the Gulf war, where most of the military operations were conducted during the night. The night eyes must respond to the specific portion of electromagnetic waves emitted at temperature when solar radiation is absent. A thermal observation device used in this research provides stabilized images by utilizing several techniques such as serial and parallel scanning, and digital image processing techniques, wherein the resolution of image is up to 240,000 pixels per frame, and detection wavelength is far infrared band ranging from 8–12 μm. The overall system (Model-TAS-970K) configuration used in this research is summarized in Table 1.

### 2.3. Optimal Infiltration-Route Analysis

**Figure 2.**
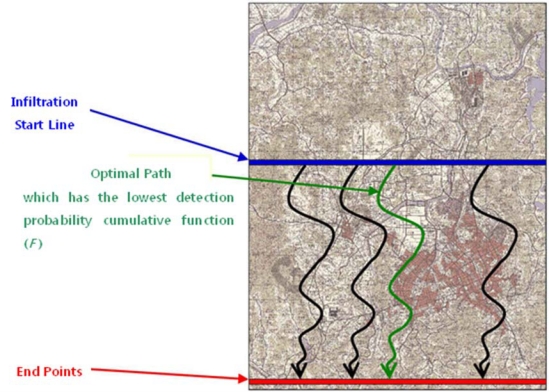
Imaginary scenario for optimal infiltration-route analysis (an imaginary MDL, Daejeon).

Infiltration is an important tactical operation in military applications, and various infiltration routes can be determined based on terrain, surveillance networks, weather, and other conditions. Figure 2 shows a scenario to determine optimal infiltration routes across an imaginary military demarcation line (MDL) in Daejeon City. The blue horizontal line illustrates the infiltration start point, and the red horizontal line demarcates the end points. Ideally, the optimal infiltration route will have the lowest cumulative detection probability function (*F*).

**Figure 3.**
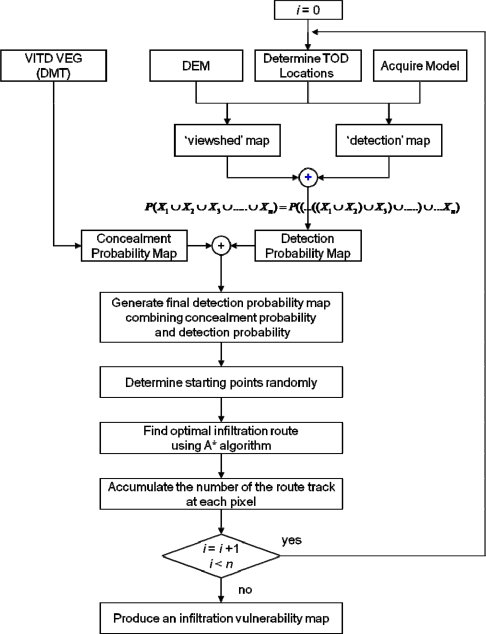
Optimal infiltration-route analysis procedure.

Figure 3 shows the overall procedure of a simulation-based infiltration-route analysis used in this research. Several layers were extracted from the vector-product interim terrain data (VITD), and they were used to compute the concealment and detection probability. A digital elevation model (DEM), which was created from contour lines on the digital maps, was used for viewshed analyses. The ACQUIRE model was used to compute the TOD detection probability. The ACQUIRE model, which has been developed by the U.S. Army Night Vision and Electronic Sensors Directorate (NVESD), is a performance assessment model for night-vision systems that can compute detection probabilities of specific targets according to distance and weather, and is based on minimum resolvable temperature differences (MRTD) [26]. Raster-based detection probability maps were created and the A* algorithm was applied to the detection probability map to determine the optimal infiltration routes while taking into consideration the terrain conditions and surveillance networks. The above operations were repeated *n*-times while the locations of the TODs and infiltration start points were randomly selected and changed. The final result is achieved by accumulating the individual optimal routing results in the form of an infiltration vulnerability map.

## 3. Simulation Design

### 3.1. Concealment Probability

**Figure 4.**
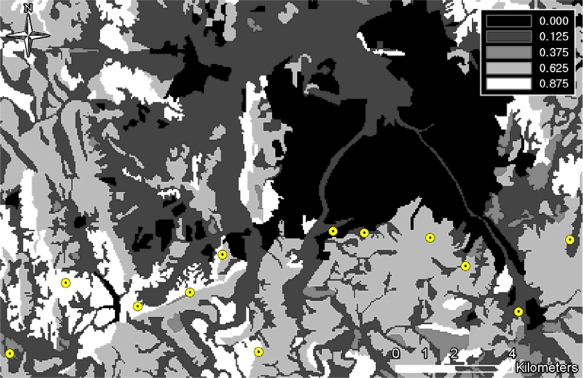
Concealment probability.

The concealment probability indicates the covering rate of infiltration with respect to vegetation and terrain occlusions. Table 2 shows the concealment probability parameters defined for the feature tables within the VEG coverage. In this study, the concealment probability was computed using three feature tables (*i.e.*, vegarea (vegetation area), vgfarea (vegetation forested area), and vgwarea (vegetation water area)); vegarea represents bushes or woods; vgfarea, from which terrain occlusion rates (DMT: Density Measure–% of Tree/Canopy Cover) can be obtained, represents forests; and vgwarea, as a hydrologic layer, determines the concealment probability based on the terrain occlusion rates. DMT represents the terrain occlusion rate caused by trees, and four different value ranges can be assigned for individual feature tables (See Table 2). In this research, the mid values of the ranges were used to compute the concealment probabilities. For the vegarea and vfwarea feature tables, the smallest DMT range values were used, since these are open-area regions. Figure 4 shows the overall concealment probability within the project site; the dark area indicates a low concealment probability, whereas the bright area represents a high concealment probability.

### 3.2. Viewshed Analysis and TOD Detection Probability

**Figure 5.**
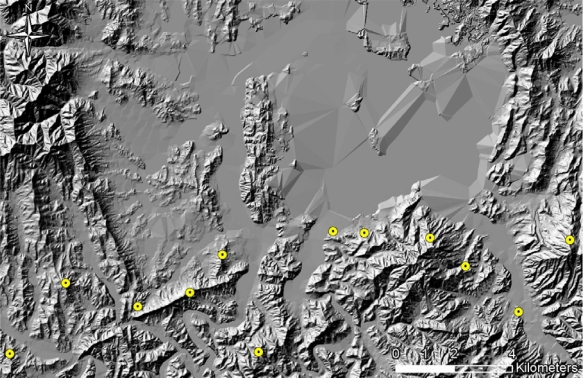
Locations of TODs.

**Figure 6.**
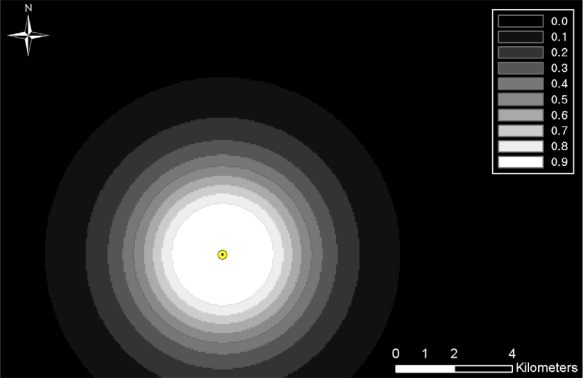
Detection probability of TOD location.

The detection probability was computed for all TOD locations, and those probabilities were then integrated with the results of viewshed analysis for the individual TOD locations. Figure 5 shows examples of TOD location. The TOD looks for invaders with a width of 0.5 m and a height of 1.5 m, and can detect objects having a 3 °C temperature difference within neighborhoods under clear weather conditions. Figure 6 shows the range of computed detection probabilities, which addresses human-size targets according to distanceusing the ACQUIRE modelunder clear weather conditions. Raster maps were created using the multiple buffer functionality in ArcGIS to represent distance-based detection probability from TOD locations. A bright tone near TOD locations indicates a high detection probability, and a darker tone farther away from TOD locations indicates a low detection probability.

**Figure 7.**
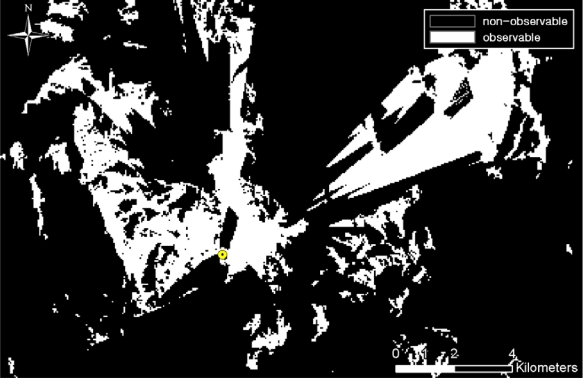
Viewshed analysis from individual TOD location.

**Figure 8.**
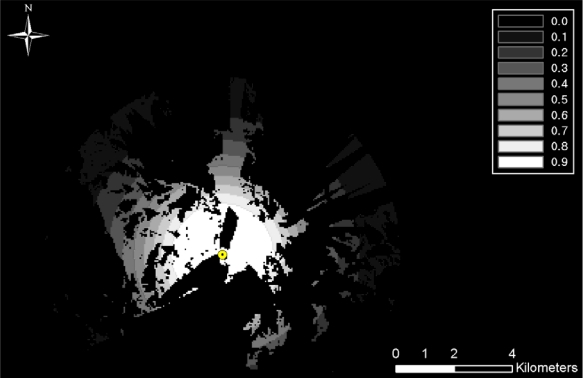
Individual TOD detection probability fused with viewshed analysis.

Viewshed analysis, using a DEM, was also performed to compute terrain-occluded regions. Figure 7 shows the viewshed analysis results from a TOD location. Observable regions from TOD locations are illustrated in white and black regions are considered non-observable. The TOD detection probability map and the viewshed analysis result map can be merged together by multiplying the two values in the corresponding cells. Accordingly, for each TOD location, the TOD detection probability and the result of the viewshed analysis were sequentially merged to compute a new TOD detection probability considering terrain conditions (See Figure 8). The detection probabilities from all of the TOD locations were merged using Equations (1) and (2):

(1) $$P\left(X_{1}\cup X_{2}\cup X_{3}\cup \cdots \cup X_{n}\right)=P\left(\left(\cdots (X_{1}\cup X_{2})\cup X_{3})\cup \cdots \right)\cup X_{n}\right)$$

(2) $$P\left(X_{1}\cup X_{2}\right)=P\left(X_{1}\right)+P\left(X_{2}\right)-P\left(X_{1}\cap X_{2}\right)$$

where *P(X_1_*∪*X_2_)* indicates the detection probability computed from at least one TOD; *P(X_1_)* and *P(X_2_)* represent individual detection probabilities from each TOD; and *P(X_1_*∩*X_2_)* represents the detection probability from both TODs.

### 3.3. Detection Probability Map

The final detection probability map was created by merging the concealment probability, *C*(*x*, *y*) (see Section 3.1), and the TOD detection probability, *D*(*x*, *y*) (see Section 3.2), where x, y represent column and row numbers, respectively, in a two-dimensional map. The TOD detection probability is positively correlated to the final detection probability, whereas the concealment probability map is negatively correlated to the final detection probability. The final detection probability is 1.0, when there is no concealment. The detection probability becomes 0 for the reverse case when the concealment is perfect (100 Accordingly the relationship between the final detection probability and the concealment probability can be written as Equation (3). Finally, Equation (4) is used to merge the two detection probabilities:

(3) P(x,y)∝(1−C(x,y))

(4) P(x,y)=D(x,y)(1−C(x,y))

**Figure 9.**
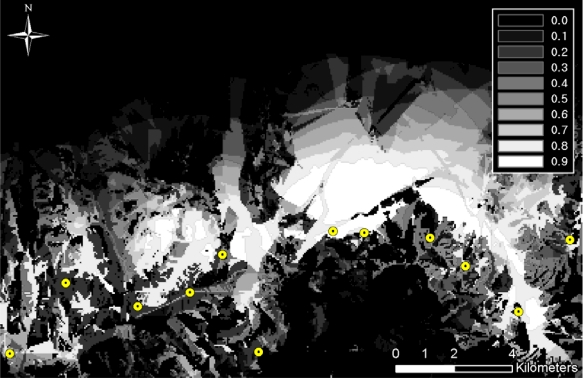
Final detection probability map.

The final detection probability map after merging the concealment probability and the TOD detection probability is shown in Figure 9. During the route analyses, the final detection probability map was considered as two-dimensional graphs with cells having 8-neighbors.

### 3.4. Dynamic Simulations

**Figure 10.**
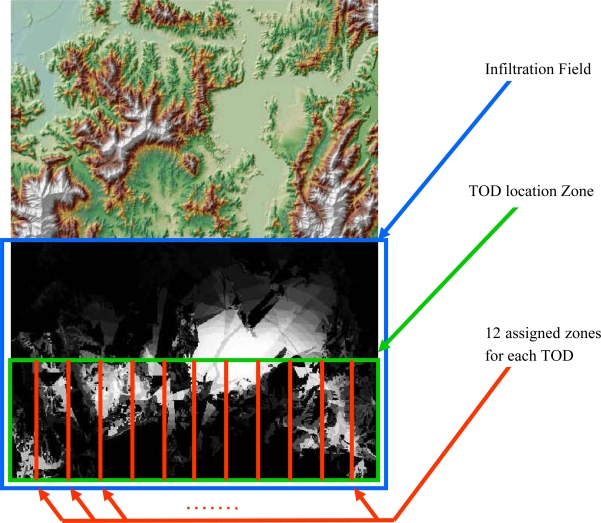
Setup for dynamic simulations.

**Figure 11.**
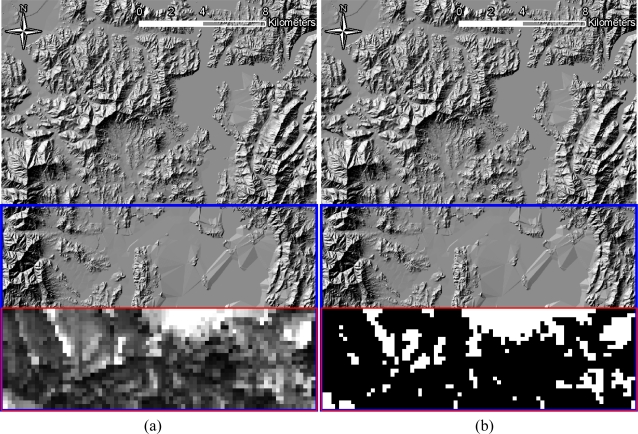
Conditions for TOD Locations. (a) The viewshed analysis result for each pixel (The bright zone represents the location of the larger viewshed in the infiltration zone). (b) The final candidate locations for TOD setups (white zone: >40% viewshed, black zone: <40% viewshed in the infiltration zone).

To this point, in building the detection probability map, the fixed locations of TODs and the infiltration start points were assumed, as illustrated in Figure 9. However, it is far from realistic to know both locations in a real-world problem, and each party can control, only, either the TOD location, in the defensive posture, or the location of the start point and the infiltration path, in the offensive posture. In order to reflect a real-world problem, the dynamic conditions of both parameters—the location of the TOD and of the start point—should be considered in the analysis. Consequently, the generation of numerous detection probability maps according to the changes of TOD location and random selections of infiltration start lines, is required. One thousand detection probability maps were produced under the assumption that the TOD location is changed. Also, twelve points on the start line of the infiltration route were randomly selected for each simulation. It would be a more comprehensive analysis if all of the points on the start line were used for the simulations; however, owing to the unmanageable computational overhead that would be incurred, that approach was not used. The TOD locations were selected under several constraints reflecting real-world conditions. The first was the location range of each TOD, which was evenly assigned in order to prevent unnecessary TOD overlap (Figure 10). The second was the condition of each TOD from the perspective of viewshed coverage—as mentioned, it is natural to locate it on the summit of a mountain or wherever it affords a wide and open view. To formulate the conditions, a viewshed analysis was applied to each pixel in the range of the TOD installation, and pixels of 40% viewshed in the infiltration zone were considered for potential TOD locations. The threshold of 40% viewshed was decided after investigating proper ranges of TOD locations in the given topography. Figure 11 illustrates the analysis and shows the TOD-location candidates.

### 3.5. Optimization Algorithms

The A* algorithm is used to minimize a cost function, *f* = *g* + *h*, where, *g* is the cost obtained from the initial node to the current node, and *h* is the estimated cost, according to the heuristic function, obtained from the current node to the goal node. In practice, if the value of *h* is 0, then the A* algorithm is equal to Dijkstra’s algorithm, which is guaranteed to find the shortest path. If the estimates of *h* are exactly equal to the real cost, then A* will only follow the best path, with minimum costs. However, if the estimates of *h* are sometimes greater than the real cost, then A* is not guaranteed to find the best path, though it can run faster than when *h* is equal to the real cost. Russell *et al.* [27] stated that the A* algorithm is effective when the heuristic function is not overestimated. Therefore, an evaluation for time complexity, completeness, and the optimality of the A* algorithm is largely dependent upon the heuristic function. Inthis research, the cost function (*g*) was defined as the cumulative detection probability from the initial node to the current node [see Equation (5)], and the heuristic function (*h*) was defined as the multiplication of the shortest distance between the initial node and the current node and the detection probability coefficient (μ*_detect_*) of corresponding cells. Thus, the optimal solution could be obtained with a smaller μ*_detect_*. In other words, by minimizing the estimates of *h*, the obtained solutions will be close to the optimal routes:

(5) $$g=\sum_{(x,y)\in A}P(x,y)$$

where A represents the collection of passed nodes:

(6) $$h=\left|y-Y_{\mathrm{goal}}\right|\times \mu_{\mathrm{detect}}$$

where *Y_goal_* is the *y*-coordinates of the end locations (see Figure 1). Thus, |y − *Y_goal_*| represents the distance from the current node to the destination line.

## 4. Experiments and Analyses

**Figure 12.**
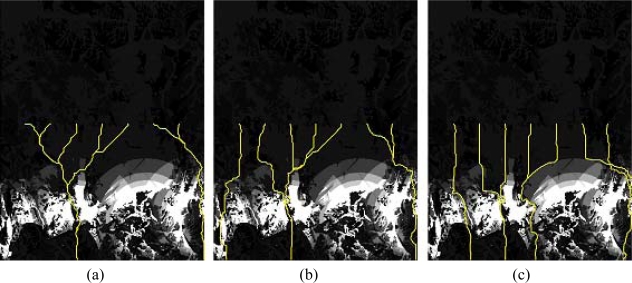
Infiltration-route results with varying μ*_detect_* values. (a) μ*_detect_* = 0.01 (b) μ*_detect_* = 0.03 (c) μ*_detect_* =0.10.

For the simulation, the standard detection probability (μ*_detect_*) value was tested and determined to be between 0.01 and 0.10 from the probability map within the project site. Figure 12 shows the path-searching results obtained using the A* algorithm with varying μ*_detect_*. values. The patterns illustrate the effects of the detection probability: the routes, down to the infiltration field, become straighter as the value becomes larger. In other words, the remaining distance from the current node to the destination has a strong influence on the computation of the total cost function. The shortest path tends to run south with an increasing cumulative detection probability. As only twelve TOD points were selected from the infiltration start line for each simulation, the tendency towards straight routing would inhibit the discovery of vulnerable pixels in the infiltration field. For that reason, the value of 0.01 was chosen for μ*_detect_*. for the remaining simulations.

**Figure 13.**
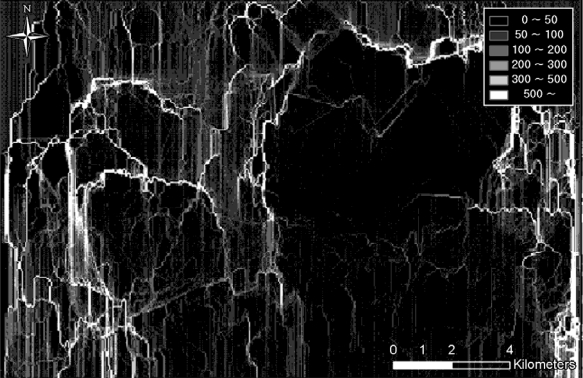
Cumulative paths of optimal infiltration routes (from 1,000 simulations).

Figure 13 illustrates the cumulative optimal route result - an infiltration vulnerability map—generated from 1,000 simulations. As mentioned, each simulation had twelve TODs at randomly selected locations, the infiltrations starting from twelve randomly selected points on the infiltration start line. Consequently, a total of 12,000 infiltrations were simulated, the optimal routes were overlapped to build an infiltration occurrence map. The Figure shows the vulnerable pixels, that is, those at which there were a high number of infiltration occurrences, in a brighter color. The result presents a clear pattern of infiltration vulnerability in mountainous areas where detection probability is generally reduced due to topographic occlusions and dense vegetations.

**Figure 14.**
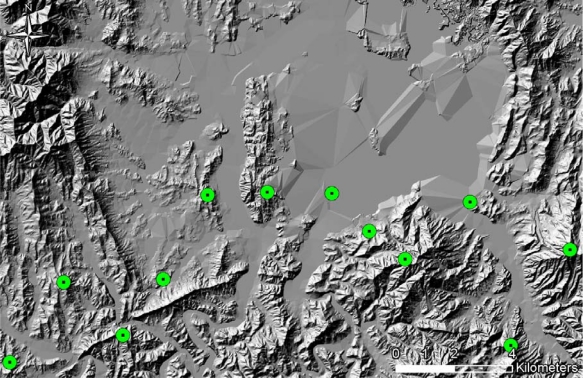
Optimal locations of TODs for vulnerable infiltration routes (from 1,000 simulations).

The value of the infiltration vulnerability map (Figure 13) can be substantiated with additional analysis for the design of a defense strategy. In order to accomplish an optimal anti-infiltration TOD defense network, TODs should be located at the positions where the total summed detection probability in the infiltration field is maximized with the vulnerability weights (*i.e.*, infiltration occurrences). In other words, by maximizing the function *k* of the following equation, the TOD locations can constitute the optimal defense network:

(7) $$k=\sum_{(x,y)\in S}O(x,y)P(x,y)$$

where *S* stands for the infiltration field, *O*(*x*, *y*) is the infiltration occurrence at the given position (*x*, *y*) according to the infiltration vulnerability map, *P*(*x*, *y*) is same as the Equation 4 which can be regenerated while moving the TOD locations. An additional 1,000 simulations were conducted, in which 1,000 different TOD-location setups were randomly generated under the same constraints described in Section 3.4 and shown in Figure 11. The TOD setup having the maximum value *k* was considered the optimal TOD network defense design. Figure 14 shows the optimal TOD locations for the vulnerable infiltration routes, with the corresponding detection probability map in the background.

## 5. Summary and Conclusions

For tactical military operations, infiltration-route analysis can provide ground infiltration routing in an attack mode, and can be used to determine the locations of defensive surveillance system equipment and guards. This research presented an optimal routing technique that determines, by dynamic simulations, potential infiltration routes in a GIS environment. Raster-based detection probability maps were created by geospatial analyses, including multiple buffering, 3D viewshed analysis, raster processing, and overlaying for the computation of concealment and detection probabilities. After building a cost map according to the final detection probability of each cell, the A* algorithm was applied to determine the optimal infiltration route by minimizing the objective function - summed detection probability on the route. This simulation process was repeated under different conditions, and an infiltration vulnerability map—showing infiltration occurrences—was created. All of the processes were conducted in the GIS environment. The value of dynamic simulation in the GIS environment was also demonstrated with another round of simulations for optimal TOD locations under the same constraints.

For more realistic infiltration analysis, additional computationally intensive simulations can be conducted. Simulations of 1,000 iterations are far from sufficient to claim ‘optimal’ route analysis, though they are probably more than enough to confirm the feasibility of the solution framework. Also, for a more realistic analytical result, meticulous design of constraints such as the number and condition of TODs as well as infiltration moving patterns and behavior, would be required.

The proposed technique, dynamic simulation in the GIS environment, can also provide comprehensive spatial analysis results for selecting optimized sites or routes for infrastructure facilities (e.g., roadways, railways, power-lines and oil/gas pipelines) or structures (e.g., bridges, power stations, *etc.*). Such dynamic simulations with spatial analyses would be feasible in the GIS environment, and the solution framework could be used, with minor modifications, for other applications, such as optimizing horizontal highway alignments. The means of locating civil infrastructures, based on terrain and other constraints, is always an important issue and dynamic simulation in the GIS environment can provide a feasible solution to this matter.

## Figures and Tables

**Table 1.** The system (Model-TAS-970K) configuration.

**Operational frequency**	far infrared(8∼12 μm)
**Scanning method**	1st generation serial-parallel scanning
**Magnification**	3∼10 × duplex magnification infrared optic
**Resolution**	0.24 mega pixels
**Monitor**	9 inch
**Focusing range**	30 m∼infinity
**Tripod height**	47 cm∼104 cm
**Operation**	directly/indirectly/vehicle mounted
**Field of view**	3 × (6.3° × 10.1°) in wide mode
	10 × (2.0° × 3.1°) in narrow mode
**Power consumption**	DC 24 V ± 6 V (direct operation)
	AC 90 V∼245 V (remote operation)
**Spinning angle**	horizontal: 360°, vertical: ± 22.5° (direct operation)
	horizontal: 350°, vertical: ± 80.0° (remote operation)
**Rotational speed**	horizontal:1.5°/sec ∼12.0°/sec, vertical: 0.3°/sec ∼1.5°/sec

**Table 2.** Concealment probability parameters.

**Layer**	**vegarea**	**vgfarea**	**vgfarea**	**vgfarea**	**vgfarea**	**vfwarea**
**DMT (%)**	-	0–25	25–50	50–75	75–100	-
**Concealment probability**	0.125	0.125	0.375	0.625	0.875	0.125
